# Three Decades of Interferon-β in Multiple Sclerosis: Can We Repurpose This Information for the Management of SARS-CoV2 Infection?

**DOI:** 10.3389/fimmu.2020.01459

**Published:** 2020-06-18

**Authors:** Martina Severa, Cinthia Farina, Marco Salvetti, Eliana Marina Coccia

**Affiliations:** ^1^Department of Infectious Diseases, Istituto Superiore di Sanità, Rome, Italy; ^2^Institute of Experimental Neurology (INSpe), Division of Neuroscience, IRCSS San Raffaele Scientific Institute, Milan, Italy; ^3^Center for Experimental Neurological Therapies, Sant'Andrea Hospital, Department of Neurosciences, Mental Health and Sensory Organs, Sapienza University, Rome, Italy; ^4^Istituto Neurologico Mediterraneo (INM) Neuromed, Pozzilli, Italy

**Keywords:** type I IFN, antiviral-activity, multiple scleorsis (MS), immunoregulation, SARS-CoV-2

## Introduction

While waiting for a vaccine, therapeutic alternatives to slow or stop the ongoing COVID-19 pandemic caused by the newly emerged severe acute respiratory syndrome coronavirus 2 (SARS-CoV2) are urgently needed. Given their wide and unspecific antiviral and immunoregulatory properties, Interferon (IFN)-α and β are among the many drugs under evaluation all over the world for their repurposing potential in this context (1). They are presently tested in clinical trials at different dosages and routes of administration in combination with other compounds such as Remdesivir, Lopinavir, and Ritonavir; chloroquine and hydroxychloroquine (2, 3) were also tested before trials halted due to safety concerns (4, 5). Many *in vitro* and *in vivo* studies in the CoV field indicate that IFN-β1a and IFN-β1b are more potent than an IFN-α subtype in the inhibition of SARS-CoV and MERS-CoV replication (2). Very recently, type I IFN's ability to suppress SARS-COV2 infection in Vero cells was also reported (6).

IFNs have been classified into three types based on their receptor usage: in humans, type I IFN contains 13 IFN-α, -β, -ω, -ε, and -κ; type II IFN includes a single IFN-γ; and type III IFN consists of IFN-λ1, -λ2, and -λ3. The effects of IFN are mediated through the induction of around 2,000 IFN-stimulated gene (ISG) products, the expression of which is mainly regulated by the JAK/STAT pathway(s). At the cellular and systems levels, in addition to their definitive antiviral and antibacterial effects, IFNs regulate, through the induction of several ISG, cell proliferation, cell cycle, survival/apoptosis, cell differentiation, and migration. While type II IFN, i.e., IFN-γ, whose expression is dramatically increased in MS, is linked to activation and maintaining of inflammation, type I IFNs (mainly IFN-αs and IFN-β) are abundantly secreted in response to viral infection, acting early during the immune response to potentiate antiviral responses and to prime and maintain adaptive immunity (7). Multiple recombinant IFN-α and IFN-β formulations have been clinically approved all over the world.

Here, we briefly review the knowledge acquired in the last 27 years of IFN-β usage for the treatment of relapsing-remitting forms of multiple sclerosis (RRMS) to hypothesize the impact of this treatment on COVID-19.

## Alteration of Type I IFN System in MS

Normally, low amounts of constitutive type I IFN accumulate in the tissue in absence of infection to support cell priming and responses to other cytokines, antiviral, and antitumor immunity, and immune homeostasis. The perturbation of this so-called “tonic,” the constitutive production of IFN (8), has been linked to the development of a number of different autoimmune diseases, including systemic lupus erythematosus, Sjögren's syndrome, or type I diabetes mellitus, often correlating with increased disease severity. Additionally, in MS, evidence on low serum levels of IFN in most patients (9) poses the basis for IFN use in MS treatment and paves the way for the investigation of endogenous defect of IFN signaling in MS. In line with this observation, the presence of SNPs and rare variants in genes involved in type I IFN signaling and antiviral pathways, namely IRF-8, STAT3, SOCS1, TYK2, ZC3HAV1, and OAS1, which may display transcriptional dysregulation in blood cells at distinct MS stages (10), were found to be altered by several published GWA studies in MS, confirming the hypothesis that an alteration of IFN-regulated antiviral responses could be linked to MS pathogenesis (11–13).

The efficacy of IFN-β, the first approved therapy for RRMS whose mechanisms of action, only partially understood, appears to be mainly related to its multifaceted pleiotropic effects resulting in sustained broad anti-inflammatory action (7), may also rely on the capacity to rescue these fine endogenous molecular defects.

In spite of the identification of MS-associated genetic alteration linked to the IFN system, only a few genomics studies have addressed in detail whether these pieces of evidence could be also highlighted at the transcriptional level in therapy-free MS patients compared to healthy donors (14). Most information on IFN gene signatures in MS has, indeed, identified them mainly as IFN-β treatment-related biomarkers in human peripheral blood mononuclear cells (PBMC) of MS patients analyzed before and after IFN-β therapy, e.g., in Malhotra et al. (15) and other references. Only recently it was shown that some ISG found expressed in PBMC or CSF of MS subjects specifically cluster with other indicators of inflammation with clinical and sex parameters of analyzed patients (16). Thus, to better understand whether dysregulation of IFN-regulated genes and pathways may be related to MS development and maintenance, we performed a systematic analysis of datasets from transcriptomes obtained from more than 400 human PBMC at distinct MS stages as well as CNS tissues and encephalitogenic CD4 T cells derived from the murine model of MS. These data indicate impaired ISG transcription profiles especially in the RR form of disease and myelin-reactive T cells and identifies a core of 21 transcripts concordantly dysregulated in all MS stages (17). In addition, recent evidence highlights dysregulations in endogenous IFN system in specific immune cell subsets, critically impacting on immune functions (18). In particular, paired analysis of B cells and monocytes from sex and age-matched control and treatment-naïve MS subjects underlined several altered previously uncharacterized ISG and pathways in MS. Notably, this study describes for the first time that expression of several ISG strictly involved in antiviral responses is strongly reduced in MS B cells and involves a profound multi-level defect in type I IFN pathway due to the low level of IFN receptors, weak STAT1 and 2 expression, and activation of and selective impairment in responses to type I but not type II IFN (18). B cells, particularly the memory subset, are human reservoirs of Epstein-Barr virus (EBV) infection. The contribution of EBV to MS pathogenesis is still being fervently debated; however, its epidemiological association with MS is clear. In line with this view and with our results pointing to an anti-viral failure, *in vitro* infection of MS B cells with EBV highlights that these cells display an altered EBV expression program and propagation, resulting in reduced containment of its infection in MS. Importantly, *in vitro* and *in vivo* exposure to IFN-β potentiates type I IFN signaling machinery in MS in turn, activating the antiviral responses and reducing the frequency of EBV-infected and proliferating B cells in MS but not healthy cultures (18).

In MS, different therapeutic strategies targeting memory B cells, including B-cell-depleting therapy, significantly reduce disease activity. The basis for this effect appears to be related to decreased production of pro-inflammatory cytokines or reduced antigen presentation by these cells. Importantly, we have reported that IFN-β therapy mediates a marked and specific reduction of memory B cells in peripheral blood of treated MS patients via a mechanism requiring a FAS-R-mediated caspase-3-dependent apoptosis, and this memory B-cell decrease is associated with reduced expression of the latent EBV gene *LMP2A* in PBMC of MS patients under IFN-β treatment (19). Altogether, this evidence points to a double-face scenario for IFN-β efficacy in MS treatment, combining anti-inflammatory and immunomodulatory actions with marked antiviral properties (20). Another important aspect in these pandemic times is that the decrease in the CD27^+^ memory B-cell compartment correlates with the concomitant increase in the CD27^−^ naïve cells, likely as a result of a renewal of circulating B cells in the peripheral blood of MS patients, offering opportunity for expansion of new virus-specific clones of antibody secreting plasma cells (19). Accordingly, *in vitro* stimulation with a TLR7 ligand (simulating viral RNA) promotes IgM and IgG production in PBMC cultures derived from IFN-β-treated MS patients as compared to the same individuals before therapy (21).

## What IFN-β Usage in MS Would Teach US in The COVID-19 ERA

International recommendations on immunization of MS patients do not indicate a specific risk for vaccination of these individuals or an increased risk for future MS development for those who vaccinate [reviewed in (22)]. Furthermore, while the recommendation does outline that patients treated with some MS therapeutics, such as fingolimod, glatiramer acetate, mitoxantrone, and rituximab, have lower responsiveness to influenza vaccination, many trials indicate that IFN-β-treated MS patients achieve significant responses and comparable protection to non-treated patients and healthy controls (22). Hence, despite the lower vaccination response rate under some treatments, MS patients contribute to the overall herd immunity toward common vaccine-preventable diseases, including (hopefully soon) a COVID-19 vaccine.

In this context, the combined properties of IFN-β as antiviral and immunoregulatory molecule could be exploited in the pandemic Phase 2 when a protective humoral immunity is desired to limit SARS-CoV2 re-infections and also from the perspective of later phases of COVID-19 management when a vaccine will be available. This key consideration implies better preservation of a functionally active and protective B-cell-mediated humoral immunity. Thus, by replenishing MS immune system with IFN-regulated functions, IFN-β therapy, alone or in continuous or cyclic combination regimen with other drugs (2, 23), may represent a treatment that combines safety and efficacy in the COVID-19 era.

## Discussion

Efforts are ongoing to understand the effects of disease modifying therapies, including IFN-β, on the risk and severity of COVID-19 in persons with MS (24, 25). Although these preliminary data are still insufficient to draw firm conclusions, they have not, so far, exhibited signals of overt danger (24, 26). This case series will soon reach the sample size that is needed to provide reliable answers to persons with MS. At the same time, it could be also verified whether IFN-β or other treatments may exert some protection against SARS-CoV2. Interestingly, an impaired IFN-α2 production in about 20% of critically-ill COVID patients has been recently described, indicating that a defective innate immune response may be associated with a poor outcome and, thus, suggesting that the timing of IFN exposure may be critical to control the virus replication and limit immune-pathogenesis (27). Further studies are required to overcome the limitation of this study, given the small number of included patients and the technical difficulties for IFN-β and IFN-λ detection, as well as to define individual genetic susceptibility that could be predictive of a molecular target for novel therapeutic strategies and treatments (27). However, in line with this view, a recent paper by Blanco-Melo et al. highlighted an unbalanced inflammatory response characterized by a reduced IFN-I and -III response to SARS-CoV-2 coupled to elevated chemokines and high expression of IL-6 in cell and animal models of SARS-CoV-2 infection and in transcriptional and serum profiling of COVID-19 patients (28). Collectively, these data provide a new and dynamic view of COVID-19-related immunopathogenic features that should be taken into consideration to pinpoint and adjust new immunomodulating therapeutic strategies.

At present, we can conclude that IFN-β remains an option in the treatment of MS, particularly during this difficult pandemic period. Ongoing clinical trials in COVID-19 and the growth of clinical data collections in MS will tell us, hopefully soon, whether this evergreen molecule may have a new role in COVID-19 treatment.

## Author Contributions

MSe wrote the article and contributed to the discussion. CF and MSa revised the article and contributed to the discussion. EC wrote the article, proposed the subject, and organized the article.

## Conflict of Interest

MSe, CF, EC received grant support from FISM related to the work. MSa received research support and consulting fees from Biogen, Merck, Novartis, Roche, Sanofi, Teva.
